# A penalized linear mixed model for genomic prediction using pedigree structures

**DOI:** 10.1186/1753-6561-8-S1-S67

**Published:** 2014-06-17

**Authors:** Can Yang, Cong Li, Mengjie Chen, Xiaowei Chen, Lin Hou, Hongyu Zhao

**Affiliations:** 1 Department of Biostatistics, Yale School of Public Health, New Haven, CT 06520, USA; 2 Program in Computational Biology and Bioinformatics, Yale University, New Haven, CT 06520, USA

## Abstract

Genetic Analysis Workshop 18 provided a platform for evaluating genomic prediction power based on single-nucleotide polymorphisms from single-nucleotide polymorphism array data and sequencing data. Also, Genetic Analysis Workshop 18 provided a diverse pedigree structure to be explored in prediction. In this study, we attempted to combine pedigree information with single-nucleotide polymorphism data to predict systolic blood pressure. Our results suggested that the prediction power based on pedigree information only could be unsatisfactory. Using additional information such as single-nucleotide polymorphism genotypes would improve prediction accuracy. In particular, the improvement can be significant when there exist a few single-nucleotide polymorphisms with relatively larger effect sizes. We also compared the prediction performance based on genome-wide association study data (ie, common variants) and sequencing data (ie, common variants plus low-frequency variants). The experimental result showed that inclusion of low frequency variants could not lead to improvement of prediction accuracy.

## Background

Genomic prediction is an important problem in genetics. It aims at predicting a phenotype outcome based on information from genetic markers, population, pedigree structures, and other relevant covariates. Recent studies suggest that genomic prediction based on genome-wide case control data (unrelated individuals) has limited prediction accuracy [1]. First, the difficulty may be caused by the polygenicity of complex traits, that is, many markers with small effects jointly affect the trait [2-4]. A larger sample size is needed to estimate those small effects more accurately. A larger sample size also leads to the improvement of prediction accuracy. Second, low frequency variants (minor allele frequency [MAF] ≤5%) have not been directly observed in genome-wide association studies (GWAS). The contribution of these low-frequency variants has not been taken into account in predictive models, which may result in the loss of prediction accuracy.

Genetic Analysis Workshop 18 (GAW18) provides both genotyping data and sequencing data of approximately 1000 samples from 20 pedigrees. This brings a good opportunity to evaluate genomic prediction from the following 2 perspectives:

1. How do we integrate the pedigree structures and genome-wide dense markers for evaluating the power of genomic prediction?

2. Can we improve prediction accuracy by including low-frequency variants?

Some pioneering studies suggested that integration of phenotype information from relatives and informative markers can improve prediction accuracy [5-7]. Linear mixed models (LMMs) have arisen as a useful tool for information integration in this context [8,9]. The random effects can used to model pedigree and the fixed effects can be used to include informative markers. It is difficult to use LMM when the number of fixed effects exceeds the number of samples. To overcome this difficulty, bayesian linear regression [6] and bayesian alphabet methods [7] have been proposed. Alternatively, an *L*_1 _estimation procedure has been proposed for LMM, named "LMMLasso" [8]. Very recently, this model has been applied to association mapping, where the random effects were used for population stratification correction [9]. However, it is computationally too intensive to apply either LMMLasso or bayesian linear regression to the genome-wide scale data set from GAW18. The aim of this study is to provide an efficient computational method to evaluate genomic prediction and answer the 2 questions above.

## Methods

### Basic model

Let *n *be the sample size. We consider the following LMM

(1) $$\begin{array}{ccc} \text{y} & = & \text{X}\beta +\text{G}\alpha +u+e,\\ \text{u} & \sim & N(0,\sigma_{u}^{2}\text{K),}\\ \text{e} & \sim & N(0,\sigma_{e}^{2}\text{I),} \end{array}$$

where $y\in R^{n\times 1}$ is the response vector; $X\in R^{n\times d}$ is the matrix of covariates (fixed effects), including the intercept and other covariates, such as age and gender; *β *is the vector for regression coefficients of the covariates; $G\in R^{n\times p}$ is the genotype matrix and *α *is the coefficient vector for *p *single-nucleotide polymorphisms (SNPs) (fixed effects); ***u ***is the random effect from $N\text{(0,}\sigma_{u}^{2}K\text{)}$; and ***e ***is the residual error with variance $\sigma_{e}^{2}$. Here the covariance matrix **K **is the genetic relatedness matrix that describes the pedigree structure among the individuals. The covariance matrix ***K ***can be constructed according to the known pedigree information or estimated from genome-wide SNP information.

### Penalized linear mixed model

There is a difficulty in applying the model when *d*+*p*+2>*n*,ie, the number of parameters exceeds the number of samples (*d *is the number of covariates, *p *is the number of SNPs treated as fixed effects, 2 is the number of variance components). To overcome this difficulty, we use penalized LMM to perform model selection [8,9]. Consider introducing a penalty on the coefficient *α*: P(α)≤t, where P(α) is the penalty and *t *is some constant. First, we can write down the log-likelihood of LMM (1) by integrating out ***u ***and ***e ***as

(2) $$\begin{array}{cc} L\left(\sigma_{e}^{2},\sigma_{u}^{2},\beta ,\alpha \right) & =\text{log}N\left(\text{y}|\text{X}\beta +\text{G}\alpha ;\sigma_{u}^{2}\text{K}+\sigma_{e}^{2}\text{I}\right)\\ & \;=\text{log}N\left(\text{y}|\text{X}\beta +\text{G}\alpha ;\sigma_{u}^{2}\left(\text{K}+\delta \text{I}\right)\right) \end{array}$$

where $\delta =\sigma_{e}^{2}/\sigma_{u}^{2}$. By eigendecomposition, $K=USU^{T}$. After some algebraic operations, we have

$L\left(\delta ,\sigma_{u}^{2},\beta ,\alpha \right)=-\frac{1}{2}\left(n\text{log}\left(2\pi \sigma_{u}^{2}\right)+\text{log det}\left(\text{S}+\delta \text{I}\right)+\frac{1}{\overset{2}{\underset{u}{\sigma }}}{\left(\text{y}-\text{X}\beta -\text{G}\alpha \right)}^{T}\text{U}{\left(\text{S}+\delta \text{I}\right)}^{-1}\text{U}\left(\text{y}-\text{X}\beta -\text{G}\alpha \right)\right)$ To maximize the above log-likelihood with the constraint P(α)≤t, equivalently, we may minimize the Lagrange form of the negative log-likelihood:

(3) $$\underset{\delta ,\sigma_{u}^{2},\beta ,\alpha }{\text{min}}\frac{1}{2}\left(n\text{log}\left(2\pi \sigma_{u}^{2}\right)+\text{log det}\left(\text{S}+\delta \text{I}\right)+\frac{1}{\overset{2}{\underset{u}{\sigma }}}{\left(\text{y}-\text{X}\beta -\text{G}\alpha \right)}^{T}\text{U}{\left(\text{S}+\delta \text{I}\right)}^{-1}\text{U}\left(\text{y}-\text{X}\beta -\text{G}\alpha \right)\right)+\lambda P\left(\alpha \right)$$

To optimize the penalized log-likelihood function, we adopt an alternating strategy as follows:

Step 1: For fixed *α*, we can treat y-Gα as the working response and use maximum likelihood (ML) or restricted maximum likelihood (REML) to obtain (*β*, $\sigma_{u}^{2}$, *δ*) using some recent algorithms proposed to efficiently solve the optimization, such as FastLMM [10] and GEMMA [11].

Step 2: For fixed (*β*, $\sigma_{u}^{2}$, *δ*), the problem (3) becomes

(4) $$\begin{array}{c} \underset{\alpha }{\text{min}}\left(\frac{1}{2\sigma_{u}^{2}}{\left({\text{U}}^{T}\left(\text{y}-\text{X}\beta -\text{G}\alpha \right)\right)}^{T}{\left(\text{S}+\delta \text{I}\right)}^{-1}\left({\text{U}}^{T}\left(\text{y}-\text{X}\beta -\text{G}\alpha \right)\right)\right)+\lambda P\left(\alpha \right)\\ =\underset{\alpha }{\text{min}}\frac{1}{2\sigma_{u}^{2}}{∥\tilde{\text{y}}-\tilde{\text{G}}\alpha ∥}^{2}+\lambda P\left(\alpha \right) \end{array}$$

where $\tilde{\text{y}}={\left(\text{S}+\delta \text{I}\right)}^{-\frac{1}{2}}{\text{U}}^{T}\left(\text{y}-\text{X}\beta \right)$ and $\tilde{\text{G}}={\left(\text{S}+\delta \text{I}\right)}^{-\frac{1}{2}}{\text{U}}^{T}\text{G}$. When we choose $P\left(\alpha \right)={∥\alpha ∥}_{1}$, the optimization problem (4) becomes the standard Lasso problem [12]. In fact, we may have some other choices of penalties, such as the elastic net [13] and MC+ penalties [14]. Coordinate descent algorithms can be used to solve the penalized regression problem efficiently [14,15].

### Implementation details

Because the optimization problem (3) is not convex, a good initial point will help to find a better solution. We started at α=0, and used REML to initialize (*β*, $\sigma_{u}^{2}$, *δ*). As in Friedman et al [15], we used $\tilde{\text{y}}$ and $\tilde{\text{G}}$ to calculate the smallest *λ *value such that all *α *equal to zero. We denote this *λ *as $\lambda_{0}$. Then we generated a decreasing sequence of *λ *values such that $\lambda_{i+1}=\eta \lambda_{i},i=0,1,...k$, where *k *is the length of the *λ *sequence. Let ${\left(\alpha ,\beta ,\sigma_{u}^{2},\delta \right)}_{i}$ be the solution corresponding to $\lambda_{i}$. When we were solving the ${\left(\alpha ,\beta ,\sigma_{u}^{2},\delta \right)}_{i+1}$ for the regularization parameter $\lambda_{i+1}$, we always used ${\left(\alpha ,\beta ,\sigma_{u}^{2},\delta \right)}_{i}$ as the initial point. This strategy accelerates the convergence of the algorithm. Typically, we set η=0.95, *k *= 20, and choose the best *λ *value by cross-validation.

## Results

### Data set and preprocessing

For the GAW18 data set, there are 959 samples from 20 pedigrees. In our study, we considered systolic blood pressure (SBP) as the quantitative trait of interest. Among all the samples, 849 individuals had at least one blood pressure measurement. We considered the first nonmissing measurement of SBP and used its log-transformed value as the response $y\in R^{849}$, with the age at the corresponding measurement as the covariate. We included the intercept as a fixed effect, giving us $X\in R^{849\times 2}$.

For the genotype data matrix ***G***, we focused on genetic markers on chromosome 3. The reason we chose chromosome 3 was that we identified a significant signal in the gene *MAP4 *by association tests on all 200 simulated SBPs. This signal was significantly stronger than the genetic background signal. We expected our method could automatically detect it and include it as a fixed effect.

We used both GWAS data and sequencing data to evaluate the model performance. For GWAS data, we applied basic quality control (MAF >0.05, missing rate <0.05), which resulted in 33,248 SNPs for chromosome 3 (ie, $G_{gwas}\in R^{849\times 33248}$). For sequencing data, there were 602,512 SNPs for chromosome 3. We first applied the same quality control criteria and then did linkage disequilibrium pruning using the threshold *r*^2 ^= 0.9, leaving 103,020 SNPs for chromosome 3 (ie, $G_{seq}\in R^{849\times 103020}$). The matrix **K **is twice the kinship matrix which is constructed based on pedigree information.

## Results

Before we applied the proposed method for prediction, we first estimated the variance that can be explained by the pedigree structure. We used LMM to do this, and included age and intercept as the fixed effects and two times kinship matrix as the random component [2]. The estimate of explained variance was 26.98% and 18.85%, for the simulated and real phenotypes, respectively. This could be an overestimate because members within the same pedigree might share some environmental factors.

**Figure 1:**
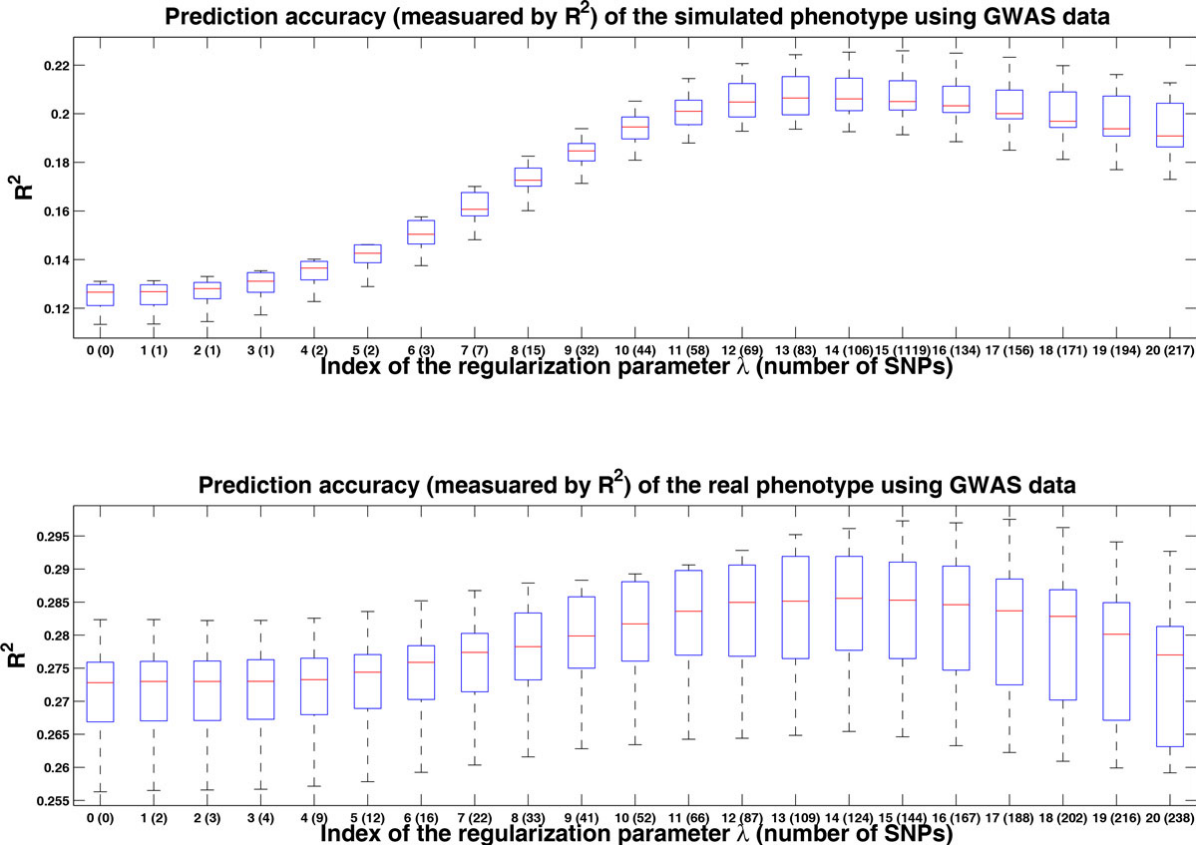
**Prediction accuracy using GWAS data**. Prediction results for the simulated phenotype and real phenotype using GWAS data. We ran 10-fold cross-validation 20 times to generate these boxplots. The x-axis is the index of the regularization parameter *λ*. The corresponding number of SNP markers used in the prediction model is given in brackets. Notice that these numbers are obtained using all samples. Index 0 corresponds to prediction only using pedigree information, thus the corresponding number of SNP marker is zero. The y-axis is the *R*^2 ^between the true value and the prediction value.

**Figure 2:**
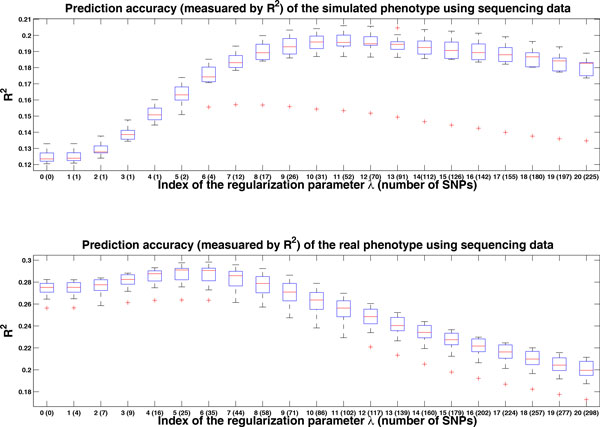
**Prediction accuracy using sequencing data**. Prediction results for the simulated phenotype and real phenotype using sequencing data. We ran 10-fold cross-validation 20 times to generate these boxplots. The x-axis is the index of the regularization parameter *λ*. The corresponding number of SNP markers used in the prediction model is given in brackets. Notice that these numbers are obtained using all samples. Index 0 corresponds to prediction only using pedigree information, thus the corresponding number of SNP marker is zero. The y-axis is the *R*^2 ^between the true value and the prediction value.

We applied penalized LMM to both GWAS data and sequencing data to evaluate its performance on phenotype prediction. Here we chose the Lasso penalty (ie, $P\left(\alpha \right)={∥\alpha ∥}_{1}$). The results are shown in Figures 1 and 2. We ran 10-fold cross-validation 20 times to generate these boxplots. We reported the result for the *λ *sequence, $\lambda_{i},i=0,1,...20$. When $\lambda =\lambda_{0}$, the model corresponds to LMM without genetic markers. As $\lambda_{i}$ decreased, more and more genetic markers were used in the model.

Based on the estimated variance components (26.98% for the simulated SBP and 18.85% for the real SBP), it seemed that we should do a better job for the simulated phenotype. From Figures 1 and 2, however, we observed that we had a better performance for the real phenotypes (*R*^2 ^= 0.205 for the simulated SBP and *R*^2 ^= 0.285 for the real SBP). The reason was that the covariate "age" contributed more for the real phenotypes. If we did prediction for both the simulated and real phenotypes without "age," we could observe a slightly better performance for the simulated phenotype.

Let us take a close look at the result using GWAS data. We used the correlation between the true value and predicted value to measure the accuracy. For the simulated phenotypes, the accuracy is around *R*^2 ^= 0.125 for $\lambda =\lambda_{0}$, and kept improving until $\lambda =\lambda_{14}$. After that, the performance started to get worse. We can see that accuracy improved from 0.125 to 0.205 as informative genetic markers were included in the model. This improvement should be mainly attributed to the simulated association signals around the gene *MAP4 *(the estimated effect size of rs11711953 is approximately −7.25 with the SE 1.46). For the real phenotype, although the performance was improved as a few genetic markers are included, the improvement was minor (*R*^2 ^increases approximately 1%). By checking the association signals, we were unable to detect significant associations.

For prediction using sequencing data, the model performed almost the same as that of GWAS data based on the simulated phenotypes. For the real phenotypes, the best accuracy achieved was close to *R*^2 ^= 0.285. Compared with the results of GWAS data, the prediction was not improved by using sequencing data.

## Discussion

In this study, we show that our model can integrate information from pedigree structures and genetic markers. This model will work well when there are a few markers with relatively large effect, as suggested by the analysis of the simulated phenotype. The reason is that the information from the pedigree structure is a global average of signals from the genetic background and the shared environmental influence. When there are some large effects that are different from the genetic background, it is better to extract them and consider them as fixed effects. In this way, the markers with larger effects can be treated locally. If all markers have similar effect sizes, the proposed method can only have minor improvement, as suggested by the analysis of the real phenotype.

In this study, we also compared the prediction performance based on GWAS data (ie, common variants) and sequencing data (ie, common variants plus low-frequency variants). The experimental result showed that inclusion of low-frequency variants could not lead to improvement of prediction accuracy. To significantly improve the prediction accuracy, the difficulty caused by polygenicity of complex traits needs to be addressed; that is, many small effects should be estimated more accurately. This implies that a larger sample size is needed. Besides the recruitment of more samples, an economic way is to combine multiple GWAS data sets of correlated traits. The underlying assumption is that these correlated traits may share some genetic factors. By borrowing information from each other, it is expected that the prediction accuracy can be dramatically improved.

Regarding the computational time, based on our current MATLAB implementation, we can finish a 10-fold cross-validation for the sequencing data in two hours.

## Conclusions

In this study, genetic prediction can be improved by combining pedigree structure and information from genetic markers. We use a penalized LMM for this purpose and we show that it is computationally feasible. The experiment result based on the GAW18 data suggests that the main prediction power comes from the pedigree information. The additional improvement could be substantial if the effect sizes of a few genetic markers are noticeable, otherwise, could be minor. Integration of multiple GWAS data sets for genomic prediction may be a promising direction.

## Competing interests

The authors declare that they have no competing interests.

## Authors' contributions

CY and HZ designed the overall study; CY, CL, MC, XC and LH conducted statistical analyses; and CY and HZ drafted the manuscript. All authors read and approved the final manuscript.
